# Multiple Idiopathic External Apical Root Resorption: A Case Report of a Rare Entity

**DOI:** 10.7759/cureus.67648

**Published:** 2024-08-24

**Authors:** Visalachi Murugappan, Roland Prethipa, Uma Maheswari T.N, Deepak Pandiar

**Affiliations:** 1 Oral Medicine and Radiology, Saveetha Dental College and Hospitals, Saveetha Institute of Medical and Technical Sciences, Saveetha University, Chennai, IND; 2 Oral Pathology and Microbiology, Saveetha Dental College and Hospitals, Saveetha Institute of Medical and Technical Sciences, Saveetha University, Chennai, IND

**Keywords:** apical root resorption, idiopathic root resorption, external root resorption, root resorption, rootless tooth

## Abstract

Idiopathic root resorption is characterized by the gradual destruction of tooth roots without a clear cause; the possible underlying factors include genetic predispositions, immune system abnormalities, or environmental influences. This case report highlights an unusual instance of a 27-year-old young female patient who presented with multiple decayed teeth; the orthopantomographic examination incidentally revealed extensive root resorptions. Thorough biochemical investigations such as acid phosphatase, alkaline phosphatase, thyroid-stimulating hormone (TSH), thyroxine (T4), and triiodothyronine (T3), as well as calcium, were within normal limits, with no identifiable local or systemic factors, leading to a diagnosis of idiopathic root resorption.

Diagnosing multiple idiopathic external apical root resorption (MIEARR) is particularly challenging, underscoring the importance of regular monitoring and comprehensive dental care to prevent further deterioration. The uniqueness of our case report lies in its investigative approach, employing advanced diagnostic techniques such as nano CT and scanning electron microscopy (SEM). It also includes a comparative analysis of cases previously reported in the literature.

## Introduction

The resorption phenomenon in hard dental structures was initially documented in the 16th century. The resorption of roots in deciduous teeth is a normal physiological process [1]. On the other hand, in permanent dentition, the etiology of root resorption involves two separate phases and is linked to a pathological base: an initial injury and a subsequent stimulus. Root resorption types are typically categorized as internal, originating from the pulp, and external, originating from the periodontal ligament [2]. External root resorption can be further subcategorized as physiological and pathological root resorption. Sometimes, diagnosing an underlying cause for root resorption can be challenging. In such cases, the condition is termed idiopathic root resorption, and it can manifest as either apical or cervical resorption. Rarely documented, idiopathic resorption of external roots can affect one or more teeth.

Mueller and Rony [3] recorded the first instance of idiopathic resorption of external roots in 1930. External root resorption can be attributed to various local and systemic factors. Locally, it may occur due to orthodontic treatment, trauma, periapical or periodontal inflammation, tumors or cysts, occlusal stress, impacted or supernumerary teeth, and dental transplantation and re-implantation. Systemic causes include endocrine imbalances, Paget’s disease of bone, renal and hepatic conditions, calcinosis, and the effects of radiation therapy. This variation's apical shape causes the tooth root to gradually shorten and rounden over time. Individuals with external root resorption typically show no clinical symptoms, though occasional complaints of tooth mobility may be reported. Diagnosing external root resorption can be challenging, as it requires a thorough evaluation of potential causes, ruling out known etiologies [4]. There are currently very few reported cases of multiple idiopathic apical resorption of roots in the literature.

## Case presentation

In accordance with the Consensus-based Clinical Case Reporting (CARE) guidelines, this report provides a comprehensive analysis of the clinical and pathological attributes of a case of multiple idiopathic external apical root resorption (MIEARR). We obtained informed consent from the patient for the use of their intraoral images and associated diagnostic workup data for research purposes.

Patient background and clinical evaluation

**Figure 1:**
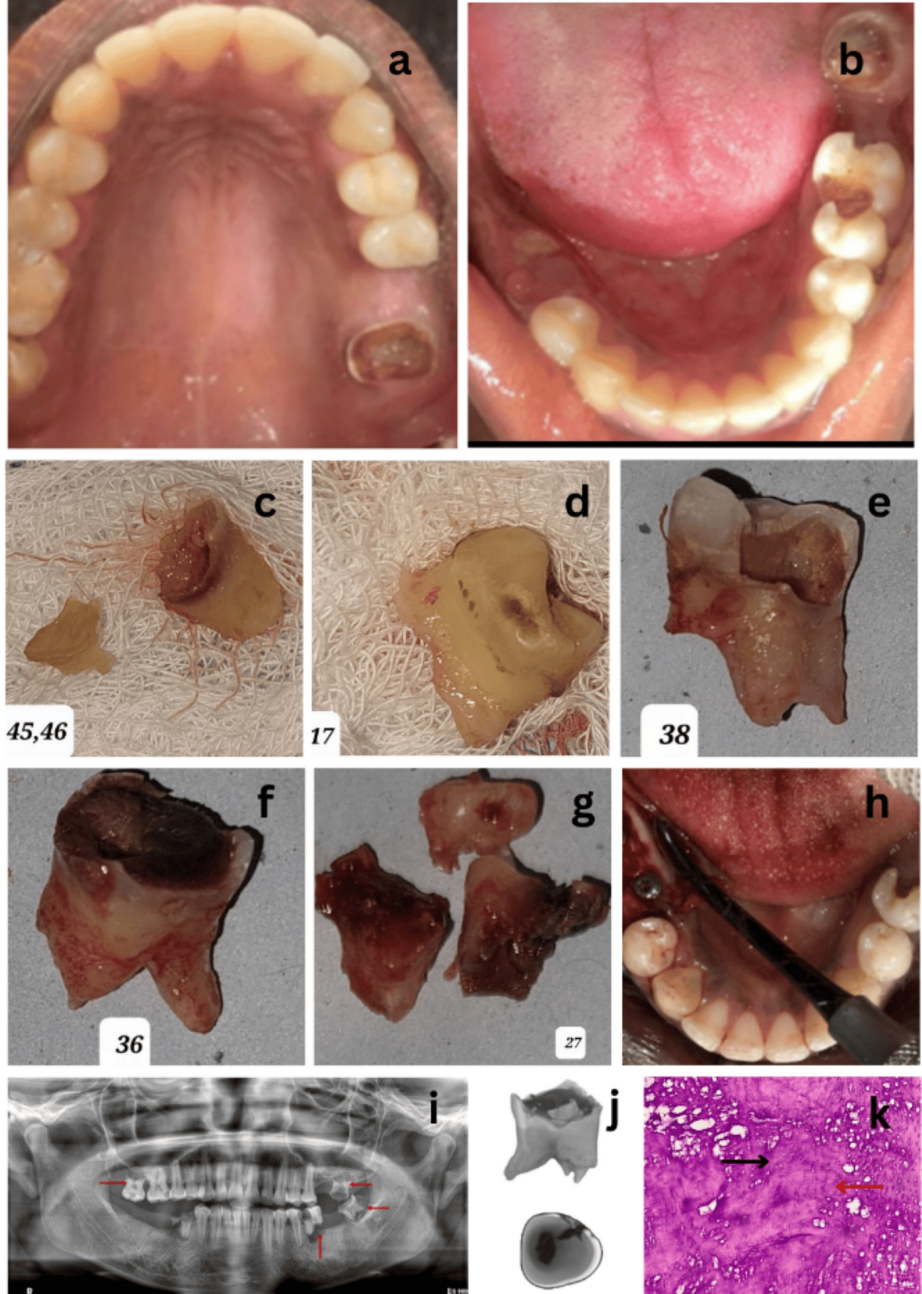
Clinical examination, investigations, and treatment procedures performed a: Maxillary intraoral occlusal view. b: Mandibular intraoral occlusal view. c: Extraction of 45,46. d: Extraction of 17. e: Extraction of 38. f: Extraction of 36. g: Extraction of 27. h: Implant placement post-extraction in 45, 46 region. i: Orthopantomographic image revealing loss of crown tooth structure in relation to 45, 46, 48 regions; missing 26, 37, 47; radiolucency involving dentin in relation to 17, 16; radiolucency involving pulp in relation to 27, 38, 36; root resorption was noted in 17, 27, 38, 36 (denoted with red arrows). j: Nano CT and SEM images of the extracted tooth. k: Photo micrograph of decalcified hematoxylin and eosin (H&E)-stained section shows wide irregularly formed dentin (denoted with red arrow), disorganized cementum (denoted with black arrow) (H&E, 10x) CT: computed tomography; SEM: scanning electron microscopy

A 27-year-old female presented to the Department of Oral Medicine and Radiology with a complaint of multiple decayed teeth in both her upper and lower arches. There was no history of trauma, and her past medical, surgical, dental, and family histories were non-contributory. Clinical examination revealed several decayed and mobile teeth (Figure 1).

Diagnostic workup

Orthopantomographic evaluation revealed multiple external apical root resorptions in the posterior teeth of both the maxillary and mandibular permanent dentition. Hematological and biochemical screening, including thyroid function tests, serum calcium and phosphorus levels, acid phosphatase, and alkaline phosphatase levels, were all within normal ranges, and hence a differential diagnosis of idiopathic external apical root resorption or a dentin dysplasia was made (Table 1).

**Table 1:** Hematological and biochemical assessment Serum calcium and phosphorus levels, as well as acid phosphatase and alkaline phosphatase levels, were within the normal range. Reference values provided in the table may vary between different laboratories T3: triiodothyronine; T4: thyroxine; TSH: thyroid-stimulating hormone

S. no	Investigations	Results	Reference ranges
1	Acid phosphatase	0.60 U/L	0.0-0.8
2	TSH	0.52 mIU/ml	0.4-4.5
3	Calcium	8.7 mg/dl	8.5-10.5
4	Phosphorus	3.1 mg/dl	2.5-4.5
5	Alkaline phosphatase	58.0 IU/L	42-98
6	T3	95.4 ng/dl	70-210
7	T4	8.0 µg/dl	5.5-11.0

The extracted teeth were preserved in 10% buffered formalin for histopathological examination, while others were preserved for evaluation using nano CT and SEM (Table 2).

**Table 2:** Tooth volume, tooth surface, and volume ratio of the whole tooth in the affected and the control group The tooth volume, tooth surface, and volume ratio of whole tooth (samples 1, 2), and enamel (samples 1, 2) were found to be lower in affected teeth when compared to the control group

Description	Unit	Whole tooth (sample 1)	Enamel only (sample 1)	Whole tooth (sample 2)	Enamel only (sample 2)	Whole tooth (control)	Enamel only (control)
Tooth volume	mm^3^	377.37	29.11	436.955	42.272	1248.59	212.27
Tooth surface	mm^2^	1122.54	599.566	1004.23	495.953	1248.83	1299.031
Volume ratio	1/mm	2.97	20.59	2.298	11.73	0.9961	6.1194

Care plan and subsequent monitoring

**Figure 2:**
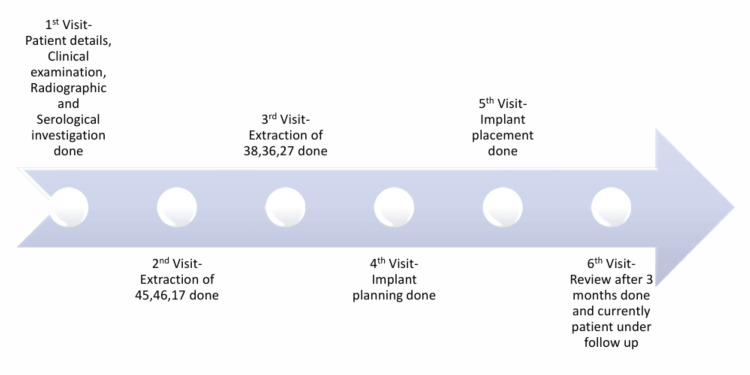
Timeline chart of the treatment according to CARE guidelines CARE: Consensus-based Clinical Case Reporting

During subsequent visits, teeth 46, 45, and 17 were extracted under local anesthesia due to decay and mobility, followed by teeth 38, 36, and 27 due to increased mobility. During the fourth visit, the possibility of dental implants for the missing teeth was discussed. Figure 2 presents a flow diagram depicting a brief timeline of the treatment from the initial visit to the follow-up.

## Discussion

The phrase "idiopathic external root resorption" was coined by Belanger and Coke to characterize situations in which root resorption occurs without apparent local or systemic reasons. Pathologic resorption is categorized into external root resorption and internal root resorption based on the lesion's location. Multiple external apical root resorption in permanent dentition can be diagnosed during routine dental examinations, and its causes are diverse as per the existing literature [4,5]. A few syndromes associated with idiopathic external apical root resorption may include Stevens-Johnson syndrome, Papillon-Lefèvre syndrome, and Turner syndrome. The term idiopathic is used when multiple external apical root resorption occurs without an apparent cause.

Typical characteristics in cases of MIEARR include clinically healthy tooth, gingiva, and periodontal tissues, symmetrical pattern of root resorption, involving both vital teeth and those that have undergone endodontic treatment, lack of inflammation in the periodontal and periradicular tissues, no evidence of alveolar bone loss or local etiological factors. Patients generally remain asymptomatic until the pathological process reaches an advanced stage, often indicated by a noticeable increase in tooth mobility. This condition is usually discovered incidentally during radiographic examinations [3,6]. A literature search for MIEARR revealed a few published case reports, with the present article contributing an additional case (Table 3).

**Table 3:** Case reports of multiple idiopathic external apical root resorption

Author	Year	Patient gender	Age, years	Teeth affected	Jaws affected	Total number of teeth affected
Cholia et al. [6]	2005	M	28	16, 17, 15, 14, 13, 12, 11, 21, 22, 23, 24, 25, 26, 27	Maxilla	14
Bansal et al. [7]	2015	M	22	16, 17, 26, 27, 36, 37, 46, 47	Both	8
Schätzle et al. [8]	2005	F	17	17, 16, 15, 14, 13, 12, 11, 21, 22, 23, 24, 25, 26, 27, 31, 32, 33, 34, 35, 36, 37, 41, 42, 43, 44, 45, 46, 47	Both	28
Soni and La Velle [9]	1970	M	34	14, 24, 25, 35, 36, 37, 45, 46, 48	Mandible	9
Cowie and Wright [10]	1981	M	27	14, 15, 16, 17, 24, 25, 26, 27, 37	Maxilla	9
Belanger and Coke [11]	1985	M	14	All permanent teeth	Both	28
Brooks [12]	1986	M	17	16, 35, 36, 46	Mandible	4
Pankgurst et al. [13]	1988	M	30	15, 16, 17, 25, 26, 28, 37, 38, 46, 47, 48	Maxilla	11
Saravia and Meyer [14]	1989	F	14	14, 15, 16, 17, 24, 25, 26, 27, 34, 35, 36, 37, 44, 45, 46, 47	Maxilla	16
Postlethwaite and Hamilton [15]	1989	M	14	11, 12, 13, 14, 15, 21, 22, 23, 24, 25, 31, 32, 33, 34, 35, 41, 42, 43, 44, 45	Maxilla	20
Rivera and Walton [16]	1994	M	24	All permanent teeth	Both	21
Di Domizio et al. [17]	2000	F	26	All permanent teeth	Both	15
Moazami and Karami [18]	2005	F	17	17, 16, 15, 14, 13, 12, 11, 21, 22, 23, 24, 25, 26, 27, 31, 32, 33, 34, 35, 36, 37, 41, 42, 43, 44, 45, 46, 47	Both	28
Khojastepour et al. [19]	2010	M	17	11, 12, 14, 22, 36, 37, 45, 46	Both	8
Kanungo et al. [20]	2013	M	16	14, 15, 17	Maxilla	3
Present study	2024	F	27	17, 27, 38, 36	Mandible	4

The reported cases suggest that MIEARR affected patients of a broad age range, from 14 to 34 years. Upon reviewing the available case reports from the literature, it was noted that a greater incidence was observed in males, with the maxilla being more commonly affected than the mandible, involving premolar and molar regions. Contrary to these findings, the current case involves a female patient with MIEARR predominantly affecting the mandible. In most cases, the mandibular front teeth were not affected. This could be attributed to the lesser vascularity of the anterior portion of the mandible compared to the maxilla. Bansal et al. [7] initially proposed a genetic predisposition in their study of a family affected with generalized root resorption. Among the cases studied, six families exhibited an inheritance pattern consistent with autosomal dominant transmission, while three families showed autosomal recessive inheritance. In contrast, our patient had no significant familial history.

Several cases of external apical root resorption do not appear to be impacted by the vascular space of the pulp. Instead, it is thought that particular stimuli could increase odontoblastic and osteoblastic activity, which would aid in the resorption of roots. Schatzle et al. [8] conducted an instantaneous histological study on removed teeth in a single instance. They noticed that the cervical portion of the root, which is covered in acellular extrinsic fiber cementum, has a considerably greater number of mineralization foci and cementicles. This result suggests a disruption in the periodontal ligament's control over mineralization.

## Conclusions

MIEARR is a complex and multifactorial condition characterized by the gradual breakdown of tooth roots without a clear underlying cause. Its diagnosis requires careful examination, often involving radiographic analysis, to identify the extent and nature of the resorptive process. Treatment options may vary based on the severity and progression of the resorption, with a focus on preserving dental function and minimizing further damage. Hence, it is essential to provide comprehensive dental care to these patients, including regular check-ups, to prevent further damage to the teeth.
